# Bayesian Population Physiologically-Based Pharmacokinetic (PBPK) Approach for a Physiologically Realistic Characterization of Interindividual Variability in Clinically Relevant Populations

**DOI:** 10.1371/journal.pone.0139423

**Published:** 2015-10-02

**Authors:** Markus Krauss, Kai Tappe, Andreas Schuppert, Lars Kuepfer, Linus Goerlitz

**Affiliations:** 1 Computational Systems Biology, Bayer Technology Services GmbH, Leverkusen, Germany; 2 Aachen Institute for Advanced Study in Computational Engineering Sciences, RWTH Aachen, Aachen, Germany; University of Kentucky, UNITED STATES

## Abstract

Interindividual variability in anatomical and physiological properties results in significant differences in drug pharmacokinetics. The consideration of such pharmacokinetic variability supports optimal drug efficacy and safety for each single individual, e.g. by identification of individual-specific dosings. One clear objective in clinical drug development is therefore a thorough characterization of the physiological sources of interindividual variability. In this work, we present a Bayesian population physiologically-based pharmacokinetic (PBPK) approach for the mechanistically and physiologically realistic identification of interindividual variability. The consideration of a generic and highly detailed mechanistic PBPK model structure enables the integration of large amounts of prior physiological knowledge, which is then updated with new experimental data in a Bayesian framework. A covariate model integrates known relationships of physiological parameters to age, gender and body height. We further provide a framework for estimation of the *a posteriori* parameter dependency structure at the population level. The approach is demonstrated considering a cohort of healthy individuals and theophylline as an application example. The variability and co-variability of physiological parameters are specified within the population; respectively. Significant correlations are identified between population parameters and are applied for individual- and population-specific visual predictive checks of the pharmacokinetic behavior, which leads to improved results compared to present population approaches. In the future, the integration of a generic PBPK model into an hierarchical approach allows for extrapolations to other populations or drugs, while the Bayesian paradigm allows for an iterative application of the approach and thereby a continuous updating of physiological knowledge with new data. This will facilitate decision making e.g. from preclinical to clinical development or extrapolation of PK behavior from healthy to clinically significant populations.

## Introduction

Providing a safe and efficacious drug therapy for large and often heterogeneous populations is a challenging objective in clinical drug development. On the one hand, a therapeutic effect of the drug is desired to be achieved for all patients; on the other hand too high concentrations have to be avoided to reduce adverse events [1, 2]. Anatomical and physiological properties have a great influence on the pharmacokinetics (PK) of drugs and lead to interindividual variability in the PK outcome [1]. However, the PK of healthy adults, which represent the natural study population in early clinical phases may differ greatly from that of the diseased target population, which is e.g. represented by elderly or children. For these populations often only sparse literature information is available about their PK behavior or physiological variability. Therefore, *in-silico* approaches are considered for the identification and characterization of sources of interindividual variability. Ideally, such approaches should be able to improve individual- and population-specific simulations of the PK of drugs and to support the knowledge-based extrapolation to other drugs or populations.

One well-known approach to characterize interindividual variability in PK parameters is nonlinear mixed effects modeling (NLME). It is a common approach to identify unexplained population variability in parameters of PK models and to identify covariates, which explain the variability of the data [3–5]. Thereby, Bayesian formulations are used for few applications, where the models are calibrated to experimental data under consideration of prior information about the parameters. The complexity of the integrated PK model varies from small and rather descriptive one or two compartment models, up to larger mechanism-based models. This allows to cover a broad range of applications, please refer to [6–8] for examples.

Another approach to describe the interindividual variability in the PK of drugs is to consider whole-body physiologically-based pharmacokinetic (PBPK) models. Whole-body PBPK models describe the PK behavior of the drug mechanistically at a very high level of detail, such that each model parameter represents an explicit physiological or substance-specific quantity [9–11]. Due to the mechanistic conceptualization, the physiologically-based compartmental model structure representing the organs is clearly separated from an underlying distribution model that quantifies the mass transfer. The distribution model itself is defined by the physicochemistry of the drug such as its lipophilicity. In particular, the differentiation of drug and physiology in combination with the mechanistic representation of human physiology allows a direct parameterization of the models. As the parameters account for a certain biological interpretation, existing large collections of prior anatomical and physiological data can be utilized from literature databases and by derivation from the physicochemical properties of the compound [12–14]. For the assessment of interindividual variability, large virtual populations are created based on such prior knowledge [15–17]. Simulations of the virtual individuals are carried out and the interindividual variability in clinical endpoints can be determined. PBPK population simulations were used in several applications e.g. for the prediction of interindividual variability in oral administration of cimetidine or for analysis of methadone distribution [18, 19]. However, the method describes interindividual variability only by use of prior information and not by an additional integration of experimental data. If only sparse prior data is available, e.g. for detailed enzymatic processes or the physiological variation in special populations, such population simulations are afflicted with large uncertainty, that cannot be reduced by further inference of information from the experimental data. Dependencies between model parameters that cannot be explained by covariates such as body mass, body height or age are difficult to integrate, since *a priori* little information may be available about such relationships.

To overcome this issue, PBPK modeling has further been used in combination with Bayesian approaches, where available prior knowledge in the form of probability distributions is updated with information extracted from experimental data in the so-called posterior distribution. Main work was done in the field of toxicokinetics [20–23], but also special applications have already been performed, recently [24–27]. Thereby, many models are case specific and therefore contain some lumped compartments or parameters to reduce complexity and dimensionality of the models. This facilitates the identification of unknown parameters but hampers the extrapolation of the results to new scenarios.

**Fig 1:**
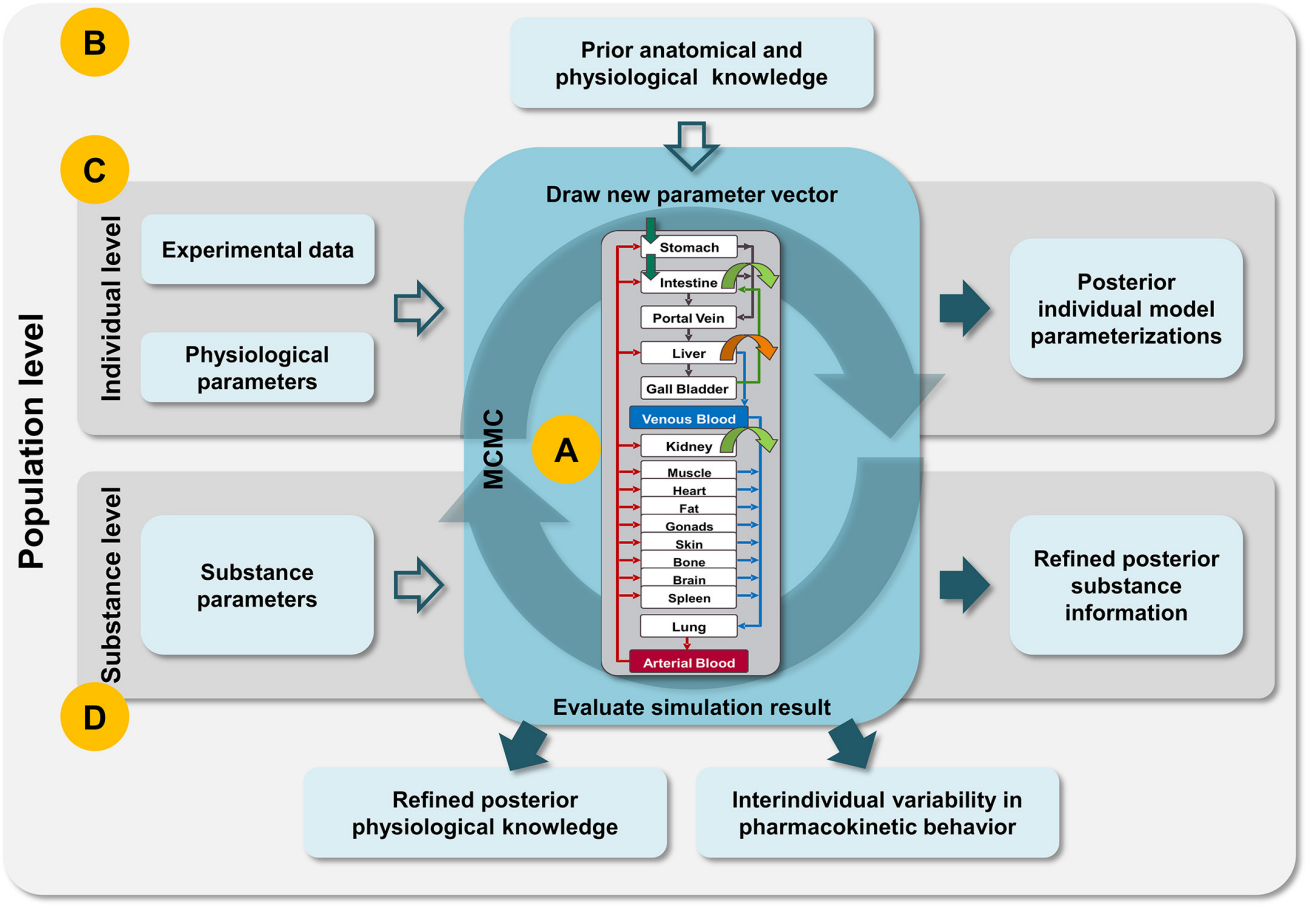
Schematic illustration of the presented Bayesian population PBPK approach. (A) A Bayesian framework is combined with a detailed mechanistic PBPK model, where a Markov chain Monte Carlo (MCMC) approach is considered to identify the high dimensional parameter distribution. (B) Prior population-specific anatomical and physiological information is integrated into an hierarchical model approach. (C) Individual-specific experimental data and physiological parameters are considered to parameterize the model and to generate individual model outputs. (D) Due to the model structure of the PBPK model, substance parameters can be differentiated from physiological parameters. This allows a global determination of the substance information, since it does not vary individually or from population to population.

In this paper a Bayesian population PBPK approach (Fig 1) is considered for the identification of interindividual variability in a population and the characterization of its physiological and ADME (absorption, distribution, metabolization, excretion)-related sources. Thereby a generic, highly detailed whole-body PBPK model constitutes the model kernel and provides a mechanistic representation of human physiology [28]. Within a Bayesian framework, a hierarchical model is incorporated to establish a separation of population level and individual level. A block-wise Markov chain Monte Carlo approach is used to identify the very high dimensional parameter distribution. In addition, a covariate model accounts for systematic variability that can be explained by the covariates age, gender and body height. For population simulations using the inferred distributions, we present a framework for estimating the posterior dependency structure and identify significant correlations of physiological parameters within the population. The integration of such correlations together with the posterior parameter distributions improves the simulation of pharmacokinetic behavior within the population compared to present approaches. In future applications, an iterative assessment of the approach could be imagined, which supports the characterization of special population’s physiology by inferring the interindividual variability over several clinical studies.

## Materials and Methods

### Physiologically-based pharmacokinetic model structure

In contrast to classical PK modeling, PBPK models describe the PK behavior of endogenous and exogenous substances in the body mechanistically. This mechanistic consideration allows a detailed representation of all important ADME processes and the individual physiology based on a large amount of prior information [9–11]. In our approach, the PBPK model is created based on a generic PBPK model structure consisting of 22 compartments including all organs, arterial and venous blood pool and the portal vein (Fig 1) [28]. Furthermore, the organs are subdivided into physiological subcompartments, which characterize the blood plasma, the red blood cells, the interstitial space and the intracellular space. All compartments and subcompartments as well as the vascular system are connected by an underlying generic distribution model that quantifies the mass transfer. The distribution model is parameterized by only few substance-specific parameters such as molecular weight, lipophilicity and protein binding. These parameters are used to determine permeabilities across membranes and partition coefficients between compartments [12–14, 29, 30]. In addition to such passive processes, active transport-, metabolization-, and excretion processes are integrated into the model based on the specific PK behavior of a substance. These processes can be represented as first or second order rate kinetics, such that also nonlinearities are considered if needed.

All individual physiological parameters such as organ volumes, blood flow rates or tissue composition are provided by the internal PBPK software database and were originally collected from large numbers of literature sources. All in all, the basic model consists of around hundred ordinary differential equations. In the model, physiological parameters are clearly separated from substance-specific parameters due to the separation of distribution model and physiological structure.

### Hierarchical modeling

Different hierarchical levels have to be considered to integrate all prior information and experimental data and to derive results both about each individual and the population (Fig 1). At an individual level, experimental PK data is provided. The PBPK model is also parameterized individually. However, prior knowledge about a physiological parameter, such as an organ volume, is provided at a population level in form of univariate probability distributions. In addition, interindividual variability needs to be derived at a population level (Fig 1).

We therefore considered a hierarchical approach consisting of two levels. The first level describes the individual PK data by the PBPK model and a proportional error model. The second level identifies the population distributions of the physiological parameters.

The model is defined as:

$$Y_{i,j}=f\left(t_{ij},\theta_{i},\nu_{i},D_{i}\right)\cdot \left(1+\varepsilon_{i,j}\right),\;\text{with}\;\varepsilon_{i,j}∼N\left(0,\sigma^{2}\right)$$ (1)

$$\theta_{i}=d\left(a_{i},\beta ,b_{i}\right),\;b_{i}∼N\left(0,I\right)$$ (2)

At the individual level in Eq (1), an experimental data point *Y*_*i*,*j*_ for individual *i* (*i* = 1…*N*) and individual specific time point *j* (*j* = 1…*n*_*i*_) at time point *t*_*ij*_ is described by function *f*, which evaluates a vector of *K* variable parameters *θ*_*i*_, a vector of fixed parameters *ν*_*i*_ and dose D_i_. Notably, the function *f* represents the mechanistic PBPK model. The proportional measurement error *ε*_*i*,*j*_ is assumed to be normal distributed with variance *σ* ^2^. In Eq (2), the variation of *θ*_*i*_ in the population is described by function *d*, which contains a vector *a*_*i*_ that represents individual characteristics like e.g. age ore height, a fixed effects vector *β* and an individual-specific random effects vector *b*_*i*_, which is assumed to be independently normal distributed and describes the unexplained variability. For a more detailed description of this well-known approach please refer to [5, 22, 31].

### Physiological covariate modeling

A large amount of physiological parameters in the PBPK model depend on the anthropometry of an individual such as its age, gender or body height [15]. The integration of such dependencies via covariates and scaling functions reduces the overall variability and in addition reduces the dimensionality of the parameter space. This allows to better identify the unexplained variability in the approach. The covariate model is defined as:

$$\begin{array}{l} a_{i}=\left(A_{i},G_{i},H_{i}\right),\\ \beta =\left(M,S\right),\\ d=M_{A_{i},G_{i}}+S_{A_{i},G_{i}}\cdot b_{i},\;b_{i}∼N\left(0,I\right)\\ d^{V}=M_{A_{i},G_{i}}\cdot {\left(\frac{H_{i}}{H_{A_{i},G_{i}}}\right)}^{\alpha }+S_{A_{i},G_{i}}\cdot {\left(\frac{H_{i}}{H_{A_{i},G_{i}}}\right)}^{\alpha }\cdot b_{i}, \end{array}$$ (3)

where the covariates *A*_*i*_, *G*_*i*_ and *H*_*i*_ represent age, gender and body height, respectively. Fixed effects *M* and *S* represent population mean values and standard deviations. Based on the structure of the appropriated physiological database [15], age- and gender-specific distributions are defined on a grid by providing mean value *M* and standard deviation *S* for each grid point. $M_{A_{i},G_{i}}$ denotes the age-scaled vector of population mean values for individual *i* specific to gender, where age scaling was performed by linear interpolation between the grid points, while $S_{A_{i},G_{i}}$ describes the age-scaled vector of population standard deviations for individual *i* specific to gender. By such formulation, the population model function *d* becomes linear. It is assumed, that the random effects *b*_*i*_ are independent from the anthropometry of an individual, which states that the unexplained variation in the parameters should be the same regardless of the covariates [5]. *A priori*, *b*_*i*_ is assumed to be independent standard normal distributed. Please find additional information below.

A specific function *d* ^*V*^ is formulated for the organ volumes, since these are also dependent on the body height [15], which is also in line with [32]. The scaling coefficient is defined as the ratio of the individual body height *H*_*i*_ and the mean height of the respective group of individuals defined by the constitutional covariates $H_{A_{i},G_{i}}$. The vector *α* represents the organ specific allometric scaling factors [15].

Notably, the population covariate model is considered for physiological parameters that are normal and lognormal distributed, respectively. In the case of a lognormal distributed parameter, $M_{A_{i},G_{i}}$ and $S_{A_{i},G_{i}}$ represent the scaled mean and standard deviation, respectively, of the associated normal distribution and the model describes *log*(*θ*_*i*_).

In summary, *θ*, *M*, *S* and *σ* ^2^ are the parameter vectors, which now need to be identified in the presented model framework to be able to describe individual-specific experimental data and assess the interindividual variability in the population.

### Separation of physiological and substance properties

Since substance information does not differ individually; it is defined as a so called “global” property of the model approach and the substance-specific parameters are identified globally for all individuals. Therefore, substance parameters in the model are considered separately from the individual parameters in the approach. This leads to *θ*_*i*_ = [*θ* ^*I*^_*i*_, *θ* ^*G*^], where *θ* ^*I*^_*i*_ represents the individual parameters, i.e. the ADME- and physiological parameters, and *θ* ^*G*^ denotes the global parameters.

### Bayesian model framework

The described hierarchical approach, the mechanistic PBPK model and all prior information is embedded into a Bayesian statistical framework. Bayesian statistics defines all unknown parameters as random variables and allows to identify probability distributions for the parameters. Thereby, parameters are inferred from existing knowledge and information contained in the experimental data [33]. In Bayes’ rule, the product of prior probability *p*(*ω*) and likelihood of the experimental data given a parameter vector *p*(*Y*|*ω*) results in the so-called posterior distribution:

$$p\left(\omega |Y\right)=\frac{p\left(Y|\omega \right)\cdot p\left(\omega \right)}{p\left(Y\right)},$$ (4)

where *Y* are arbitrary experimental data and *ω* are arbitrary parameters. A large number of parameters are integrated in the presented Bayesian population PBPK approach, which leads to a very high dimensional parameter space. Because of the high dimensionality of *ω* the determination of the scaling factor *p*(*Y*) = ∫ *p*(*ω*|*Y*)*dω* is almost impossible. Therefore, Markov chain Monte Carlo (MCMC) approaches have been developed, which describe a growing class of sampling algorithms that allow the estimation of the posterior distribution by drawing a large sample out of it. In contrast to classical Monte Carlo sampling, MCMC methods sample along a Markov chain that has the posterior distribution as its long-run stationary distribution [34]. After a so-called burn-in period which is needed to converge from an initial parameter vector to the stationary distribution, each iteration of the MCMC approach represents a parameter vector out of the posterior distribution. The bandwidth of MCMC approaches ranges from the basic Metropolis-Hastings algorithm [35] to very advanced MCMC strategies including multivariate sampling [36], adaptive MCMC [37] or Riemann manifold Langevin and Hamiltonian Monte Carlo methods [38]. A very good overview can be found in [39].

In this work, the MCMC approach described in Krauss et al. [24] is extended to assess the hierarchical model as described above. A block-wise Metropolis-Hastings (MH) approach [33, 39] copes with the different sampling of population parameters, global parameters and individual parameters. For each individual, the parameter vector containing all individual parameters *θ* ^*I*^_*i*_ is sampled in one block. The global parameters *θ* ^*G*^ are sampled together with *σ* ^2^ in another block. *M* and *S* are sampled separately and are further divided into 2 blocks.

As an exemplary MCMC step, sampling of the individual parameters of individual *i* is performed as follows:

1. Let $\theta_{i}^{I}\left(n\right)$ be the individual parameter vector of individual *i* after *n* steps. Propose a new parameter vector $\theta_{i}^{I}\prime$ by random sampling from a proposal density $Q\left(\theta_{i}^{I}\left(n\right),\cdot \right)$.
2. Generate *u* ∈ [0,1] uniformly distributed. Examine if
  $$u\leq \frac{p\left(\theta_{i}^{I}\prime |Y_{i},\theta^{G}\left(n\right),M\left(n\right),S\left(n\right),\sigma^{2}\left(n\right)\right)\cdot Q\left(\theta_{i}^{I}\left(n\right),\theta_{i}^{I}\prime \right)}{p\left(\theta_{i}^{I}\left(n\right)|Y_{i},\theta^{G}\left(n\right),M\left(n\right),S\left(n\right),\sigma^{2}\left(n\right)\right)\cdot Q\left(\theta_{i}^{I}\prime ,\theta_{i}^{I}\left(n\right)\right)}$$ (5)
3. If true: $\theta_{i}^{I}\left(n+1\right)=\theta_{i}^{I}\prime ,$ else: $\theta_{i}^{I}\left(n+1\right)=\theta_{i}^{I}\left(n\right).$

The proposal density Q is defined as truncated normal distribution centered around $\theta_{i}^{I}\left(n\right)$. The lower and upper bounds of the parameters are chosen as defined in S1 Table and are further described below. Each parameter of one block is sampled independently, such that the proposal covariance matrix becomes diagonal. The same applies to the other parameter types.

Exceptions are the organ volumes $\theta_{i}^{I,OV}=\left(\theta_{i,1}^{I,OV},\ldots \;,\theta_{i,V}^{I,OV}\right)$, which are assumed to summarize to individual’s body weight [15]. This additional constraint must be taken into account during sampling. To avoid complex transformations, the sampling process is performed under consideration of a consecutive adaption of the lower and upper bound of a certain organ volume, conditional on the remaining proportion of BW. This is performed as follows:

1. Sort $\theta_{i}^{I,OV}$ in ascending order related to the difference of the parameter-specific lower and upper bound $\theta_{i}^{I,OV,max}-\theta_{i}^{I,OV,min}$ and set *BW*_*rem*_ = *BW*.
2. Sample a new organ volume candidate $\theta_{i,v}^{I,OV\prime },v=1\ldots \;V$ from a truncated normal distribution centered around $\theta_{i,v}^{I,OV}\;(\text{n})$ with $\theta_{i,v}^{I,OV,min^{*}}<\theta_{i,v}^{I,OV\prime }<\theta_{i,v}^{I,OV,max^{*}}$, where
  $$\begin{array}{l} \theta_{i,v}^{I,OV,{min}^{*}}\;=\;max\left(\theta_{i,v}^{I,OV,min},BW_{rem}-\sum_{j=v+1}^{V}\theta_{i,j}^{I,OV,max}\right),\\ \theta_{i,v}^{I,OV,{max}^{*}}\;=\;max\left(\theta_{i,v}^{I,OV,max},BW_{rem}-\sum_{j=v+1}^{V}\theta_{i,j}^{I,OV,min}\right). \end{array}$$ (6)
3. Determine the remaining body weight $BW_{rem}=BW-\theta_{i,v}^{I,OV\prime }$.
4. Repeat steps 2. and 3. until = *V* − 1. Then set $\theta_{i,v=V}^{I,OV\prime }=BW_{rem}$.

The adaptation of lower and upper bounds allows an efficient independent sampling of each organ volume. Apart from the dynamic lower and upper bounds, the organ volumes can be treated the same way as all other individual parameters, such that the block-wise MH algorithm (see Eq (5)) is unaffected by the additional sum-constraint of the organ volumes.

The likelihood describes how well the model, which is parameterized with a certain parameter vector, describes the experimental data. The individuals are assumed to be independent from each other, such that likelihood and prior can be factorized related to the individuals.

Based on the proportional error model, the likelihood for individual *i* is defined as:

$$p\left(Y_{i}|\theta_{i}\right)=\prod_{j=1}^{n_{i}}\frac{1}{\sqrt{2\pi {\left(\sigma \cdot Y_{i,j}{}^{m}\right)}^{2}}}\cdot \text{exp}\;\left(-\frac{1}{2\sigma^{2}}\cdot {\left(\frac{Y_{i,j}-Y_{i,j}^{m}}{Y_{i,j}^{m}}\right)}^{2}\right),$$ (7)

where $Y_{i,j}^{m}=f\left(t_{ij},\theta_{i},\nu_{i},D_{i}\right)$ describes the model output for parameter vector *θ*_*i*_. A heterogeneous amount of prior information is available for the model parameters. Therefore, different types of distributions are considered according to the present knowledge. The full prior for individual *i* assuming independence between the different parameter types is specified as:

$$p(\theta_{i}^{I},\theta^{G},M,S,\sigma^{2})=\prod_{k=1}^{K}p(\theta_{i,k}^{I}|M,S)\cdot \prod_{l=1}^{L}p(\theta_{l}^{G})\cdot \prod_{k=1}^{K}p(M_{k})\cdot \prod_{k=1}^{K}p(S_{k})\cdot p(\sigma^{2})$$ (8)

The following prior distributions are considered for single parameters of the several types of parameters:

An individual parameter $\theta_{i,k}^{I}$ is assigned a prior based on the population model, assuming a truncated normal or lognormal distribution with covariate-scaled mean value and standard deviation $M_{A_{i},G_{i}}$ and $S_{A_{i},G_{i}}$, as described in the section “Physiological covariate modeling” above. The constraints for each individual parameter and the distribution type are denoted in S1 Table.

A global parameter $\theta_{l}^{G}$ is assigned a truncated uniform distribution. The constraints for each global parameter are denoted in S1 Table.

The population mean values *M* are also assigned prior distributions, called hyper priors. Each *M*_*k*_ is assigned a truncated normal distribution if the certain parameter is included into the physiological database of the PBPK modeling software. The mean value of the hyper prior is taken from the physiological database and a coefficient of variation (CV) of about 20% is assumed [40, 41]. Constraints are determined as ± 2 times the standard deviation. The intestinal permeability and active clearance processes are not included in the physiological database, such that they are assigned a truncated uniform distribution. For these parameters, constraints are defined as ± 2 orders of magnitude around the initial value.

The population standard deviations *S* are also assigned hyper priors. Each *S*_*k*_ is assigned a truncated normal distribution if the certain parameter is included into the physiological database of the PBPK modeling software. The mean value of the hyper prior is taken from the physiological database and a coefficient of variation (CV) of about 50% is assumed [40, 41]. Constraints are determined as ± 2 times the standard deviation. For the intestinal permeability and the active clearance processes the hyper prior for *S* is defined as an inverse Gamma distribution with scale parameter a = 1 and shape parameter b = 0.22, which is a standard prior for variances and standard deviations [40, 42].

The measurement error *σ* ^2^ is assigned a scale-invariant uninformative prior [43]

p(σ2)=1σ2 (9)

The result of an MCMC run is a large chain of high dimensional parameter vectors representing the posterior distribution, which contains the refined information in physiological properties like organ volumes or enzyme activity and refined substance parameters such as lipophilicity or protein binding (Fig 1). The individual- and population-specific results can then be used for individual- as well as population-specific simulations as presented in an application example below.

### Posterior population model simulations using an *a posteriori* dependency structure

As a prior assumption, we consider independent population distributions for each physiological parameter by definition of *b*_*i*_∼*N*(0, *I*) in Eq (3). However, the posterior distribution can contain dependencies between population parameters.

To estimate such dependencies and further take them into account for model simulations, we first obtain the posterior random effects of subsamples *z* = 1…*Z* of the posterior Markov chain by inverting the population function *d* of Eq (3):

$$b_{i,k,z}\;=\frac{\theta_{i,k,z}^{I}-M_{A_{i},G_{i}}^{k,z}}{S_{A_{i},G_{i}}^{k,z}},$$ (10)

where *b*_*i*,*k*,*z*_ is the posterior random effect of individual *i*, parameter *k* of subsample $z,\;\theta_{i,k,z}^{I}$ is the posterior physiological parameter of individual *i*, parameter *k* of subsample $z,\;M_{A_{i},G_{i}}^{k,z}$ and $S_{A_{i},G_{i}}^{k,z}$ are the age-, gender- (and height-) scaled posterior population mean value and standard deviation of parameter *k* of subsample *z*. We then assume a multivariate Gaussian mixture model

$$p\left(b\right)\;=\;\sum_{z=1}^{Z}\;N\left({\bar{b}}_{z},\Sigma_{z}\right),$$ (11)

where ${\bar{b}}_{z}=\left({\bar{b}}_{1,z},\ldots \;,{\bar{b}}_{K,z}\right)$ and ${\bar{b}}_{k,z}=\frac{1}{N}\sum_{i=1}^{N}\;b_{i,k,z}$ is the mean value of random effects for parameter *k* and subsample *z* of all *N* individuals and Σ_*z*_ is the covariance matrix of all random effects of subsample *z*.

A new individual parameter vector $\theta_{i,z}^{I*}$ can then be generated by consideration of a randomly chosen subsample *z*, where

$$\theta_{i,z}^{I*}=M_{A_{i},G_{i}}^{z}+S_{A_{i},G_{i}}^{z}\cdot b_{z}^{*},\;b_{z}^{*}=N\left({\bar{b}}_{z},\Sigma_{z}\right).$$ (12)

This new “individual” can then be simulated by model parameterization with $\left[\theta_{i,z}^{I*},\;\theta_{z}^{G}\right]$ and evaluation of the PBPK model.

### Software tools and computation

The PBPK model considered in this work was created with the software tools PK-Sim^®^ (version 5.3.2; Bayer Technology Services GmbH, Leverkusen, Germany) and MoBi^®^ (version 3.3.2; Bayer Technology Services GmbH, Leverkusen, Germany), for which academic licenses are available free of charge. PK-Sim^®^ contains a generic physiological compartmental structure as also described in section “Physiologically-based pharmacokinetic modeling” above. The selection of several generic distribution models using different methods for calculation of partition coefficients is possible. In this work we used the distribution model by Rodgers and Rowland [13, 14]. Based on the anthropometry of an individual, all physiological parameters like organ volumes and blood flow rates are determined using an integrated physiological database. MoBi^®^ is a tool for mechanistic and dynamic modeling of biological processes, which is compatible with PK-Sim^®^. This allows merging of several PBPK models, which e.g. describe the parent compound and its metabolites. PK-Sim^®^ and MoBi^®^ have both been explained in detail before [12, 28, 44, 45]. The full Bayesian approach including the MCMC methods and the hierarchical model structure was implemented in Matlab^®^ (version R2013b; MathWorks, Natick, MA). The MoBi^®^ Toolbox for Matlab^®^ constituted the interface to the PBPK modeling software and was used for parameterization and simulation of the PBPK model. The model output was then further processed in Matlab^®^. Notably, such toolbox is also available for the software package R such that the full model approach can also be considered in open source software.

Computation was performed on a computer cluster running the SUSE Linux Enterprise Server 11 SP3 operation system. The cluster consists of 36 knots whereby each knot consists of 2 CPU containing 16 cores. Model evaluations were parallelized such that each individual was evaluated on a single core. Running time was about 6.6 s/iteration.

### Application example: theophylline model

As an application example we here considered the PK of theophylline, a methylxanthine drug that acts against asthma and chronic obstructive pulmonary disease. Individual dosings are considered in theophylline therapy due to large interindividual variability. Thereby, the sources of variability vary widely and no clear relationship to classical covariates like age, gender or body weight can be observed. In addition, a variety of other factors influence theophylline PK such as e.g. different diseases [3, 46–48].

A free available theophylline dataset [49] was taken to demonstrate the abilities of our approach. S2 Table provides the experimental data, which is shown in S1 Fig, additionally. The data is easily accessible in R as “*theoph*” data frame. It consists of 11 measurements of the theophylline concentration in venous blood plasma for each of 12 individuals together with the administered doses and the body weights of the individuals. In addition, one data point for the fraction of unchanged theophylline in urine after 36 hours was considered [47], together with a fixed measurement uncertainty of 5%. Such additional data allows for the identification of several clearance processes such as hepatic clearance and renal clearance. An individual PBPK model was created for each of the 12 individuals. Since no age or height was stated in the dataset, it was assumed that all individuals are 30 years old but differ in body height. Body height was randomly chosen such that body mass index is in normal ranges (in between 19 and 25). This assumption indicates a very homogenous group of individuals simulating e.g. a first in man study of a new drug. S3 Table shows the anthropometry and related administered dose together with appropriated body heights. Two clearance processes were integrated into the model, hepatic metabolization via cytochrome P450 1A2 and a renal excretion process [47]. For both clearance routes a first order process was considered.

To identify reasonable start parameters for our investigation, a parameter identification process was performed for a mean value model to identify a good guess for the specific clearance rates and the intestinal permeability, which is an important parameter defining the absorption of the drug. For the drug specific parameters, which characterize the physicochemistry of the drug, literature information was used to define a reasonable start value [47]. The physiology was specified using the respective entry of the integrated physiological database of the used PBPK modeling platform PK-Sim [15, 28]. All in all, 38 parameters which define the individual’s physiology were varied in the approach for each individual, together with 2 global substance-specific parameters (S1 Table). Each individual parameter was assigned two population parameters *M* and *S*, the population mean and the population standard deviation, respectively, which were also varied in the approach. In addition, the variance of the measurement error for the venous blood samples was varied, while the variance of the measurement error for urinary excretion was set to an assumed fixed value of 5% since only one data point was available. Due to 12 individuals considered, this resulted in 535 parameters (12 individuals x 38 parameters + 2 global parameters + 38 population mean values + 38 population standard deviations + 1 measurement uncertainty) that have been identified in the approach.

Several pre-runs have been performed to adapt the proposal densities, which are critical for a good performance of the MCMC approach. After each pre-run, the proposal densities and the start values of all parameters have been adapted. The start values were defined as the last sampled parameter of the previous run. The proposal standard deviations were defined as a proportion of the standard deviation of the previous posterior sample chain.

In our final MCMC run a posterior sample of about 1,000,000 iterations was created. A burn-in period of 200,000 parameter samples was cut off, since after 200,000 iterations convergence of the posterior chain could be assumed by visual inspection and determination of the Gelman and Rubin convergence criterion [50, 51]. Since our MCMC approach consists of one long run but Gelman and Rubin’s convergence criterion requires at least two chains, we split our chain into two chains with equal length (200,001:600,000 and 600,001:1,000,000) and calculated the Gelman and Rubin convergence criterion $\hat{R}$. S4 Table shows the obtained values for $\hat{R}$ for important parameters. From the remaining 800,000 samples a subsample of 500 parameter vectors was drawn to get an independent subsample of the posterior. This subsample was then considered for the following analyses.

## Results

### Individual-specific model simulations

**Fig 2:**
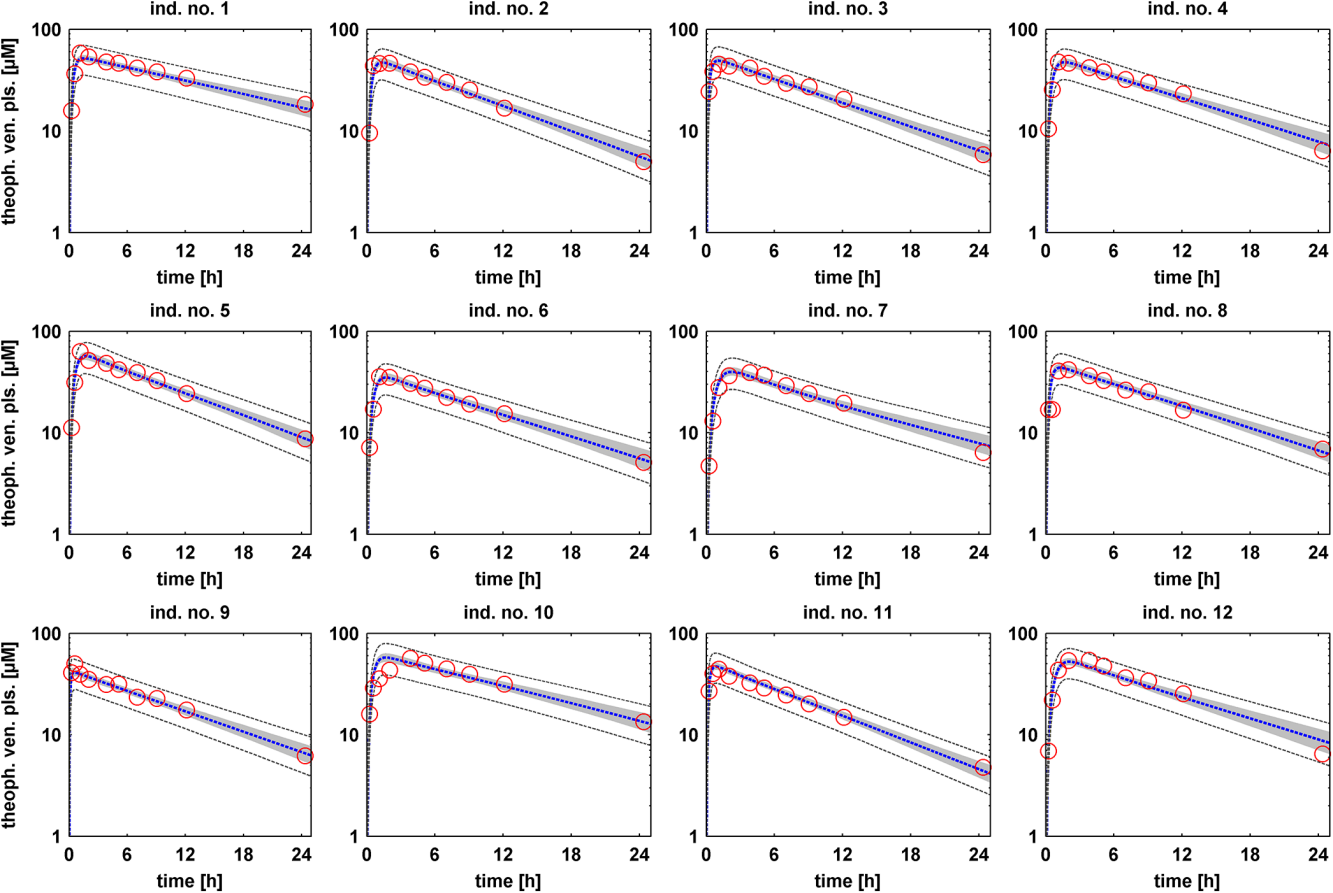
Individual-specific model simulations of theophylline venous plasma concentrations. For each of the 12 individuals the PBPK model was subsequently parameterized and simulated with each of 500 individual and independent parameter vectors out of the posterior distribution. The 95% confidence interval of all simulations (grey area) is shown together with the mean value curve (blue dotted line) and the experimental data (red circles). Dark grey dotted lines depict the upper and lower bound of the 95% confidence interval of all simulations including the inferred measurement error.

**Fig 3:**
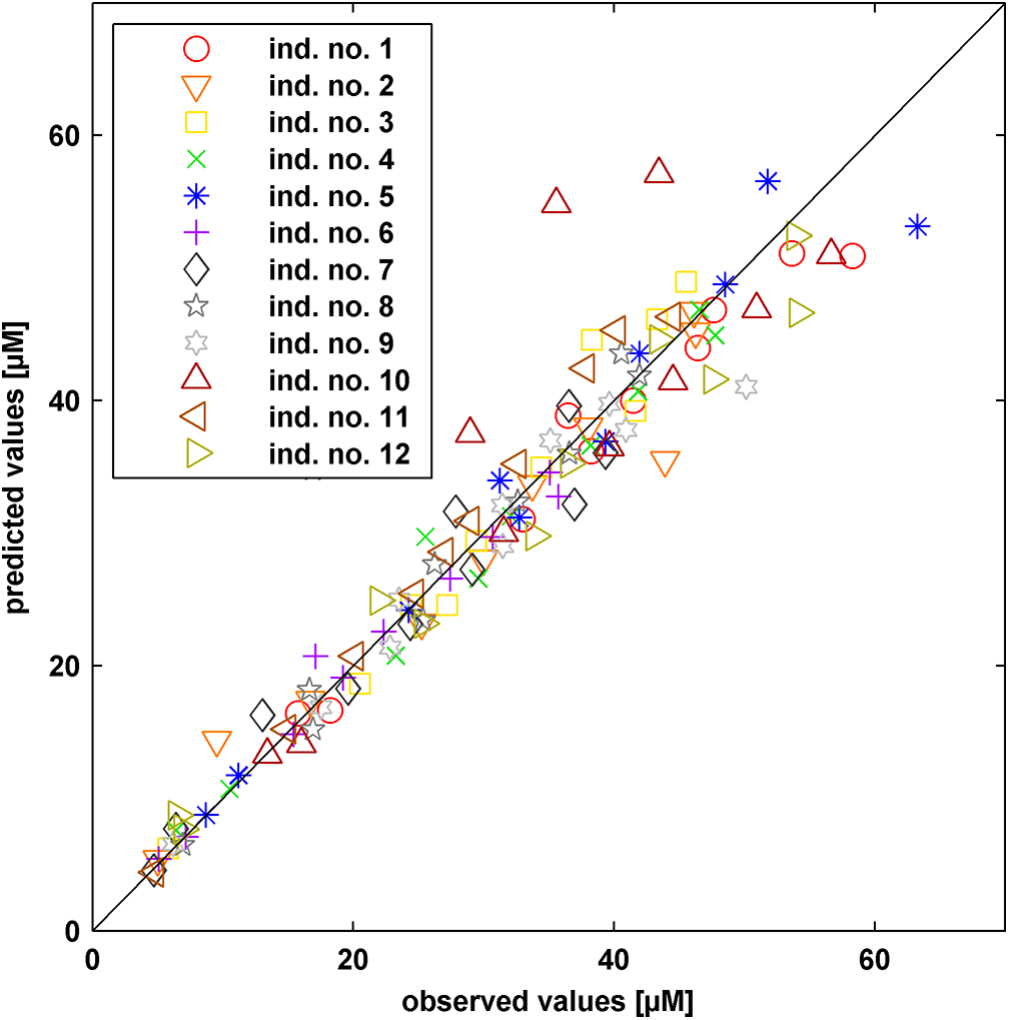
Comparison of observed experimental data and simulated values. Mean simulated values are plotted against the observed data at the same time points for all individuals (different markers, see legend in the figure).

As a verification of the individual level model and the structural properties of the PBPK model, individual simulations were performed out of the posterior sample and compared to the experimental data. Fig 2 shows the individual PK behavior for all individuals. The PBPK model was parameterized one after another with all of the 500 posterior subsamples and the PK was simulated. The 95% confidence interval of all model simulations is shown together with the experimental data and the mean value curve. Furthermore, Fig 3 shows the predicted values versus the observed data for a better visual inspection of the quality of the fit.

Except for individual no. 10, the PK behavior is described well. Individual no. 10 seems to have a much slower absorption of the drug, which can be related e.g. to a tablet that does not dissolve completely. Such effects cannot be described by our model. The confidence intervals of the PK simulations are narrow indicating only small uncertainty in the simulations. Notably, by including the measurement uncertainty into the simulations all experimental data can be fully explained by the model approach. S2 Fig additionally depicts the simulations in linear scale.

### Inference on parameter level

**Fig 4:**
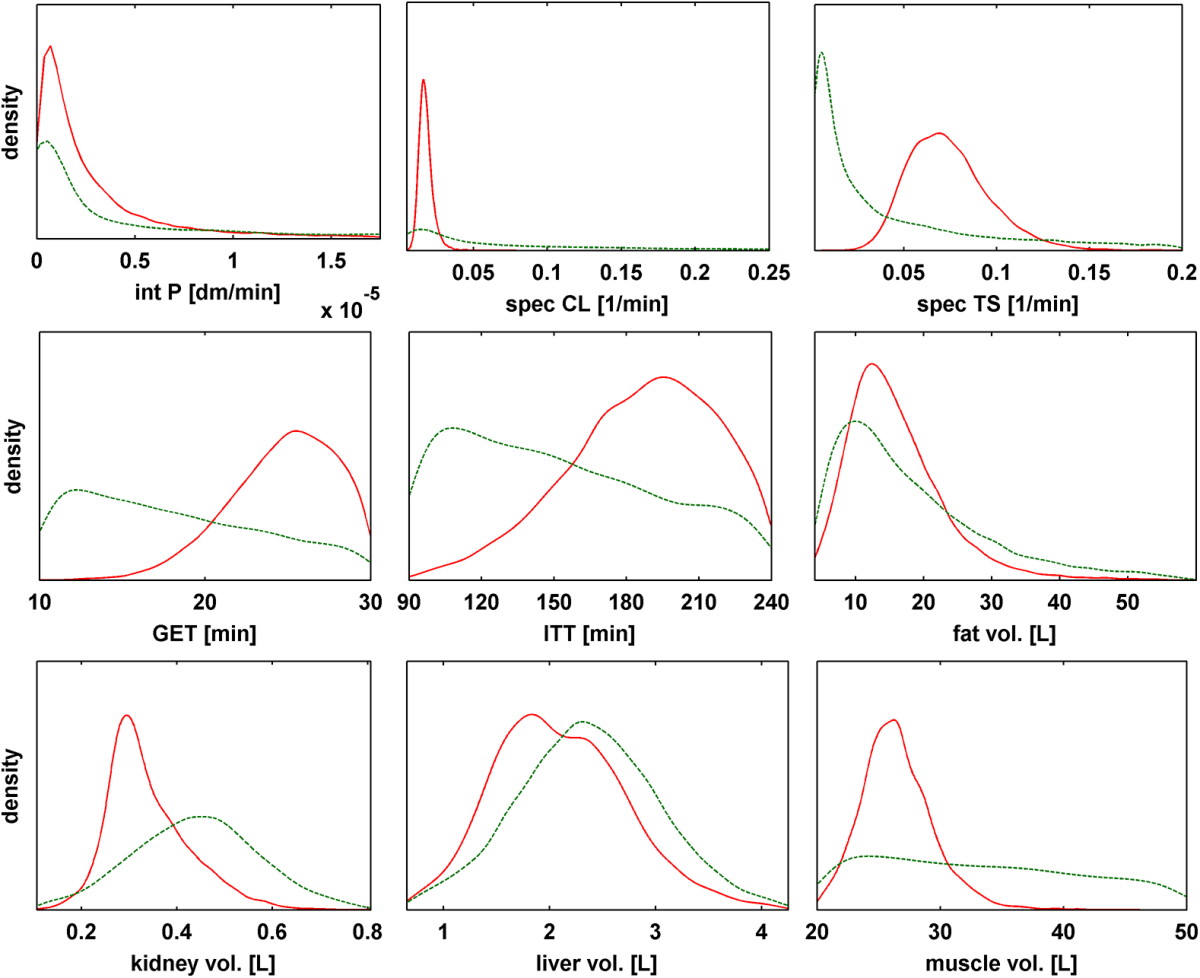
Comparison of marginal prior and posterior distributions of nine exemplary physiological parameters. For each parameter, the marginal posterior density estimate out of the full posterior (red line) is compared to the corresponding prior distribution (green dotted line). Limits on x axis represent physiological constraints as defined in S1 Table (except for intP where the maximum x value was reduced by a factor of 20 and for specCL were the maximum x value was reduced by a factor of 2 for better visualization)

The Bayesian inference that is generated with the Bayesian population PBPK approach is represented by the high dimensional posterior distribution of all parameters. Simulations and extrapolations on the PK level can only be performed by using high dimensional samples of the posterior. However, the consideration of marginal parameter distributions is an appropriate instrument to illustrate parameter inference. Fig 4 shows the marginal posterior population distributions for nine exemplary parameters: the intestinal permeability (intP), the specific hepatic clearance rate (specCL), the specific tubular secretion rate (specTS), the gastric emptying time (GET), the intestinal transit time (ITT) and four organ volumes. 10000 random iterations out of the posterior sample were considered to estimate the marginal population distributions. The uncertainty of the population mean values and standard deviations were integrated by using Eqs 11 and 12 for generation of the parameters. To account for the parameter constraints, truncated distributions were considered. The probability density function (pdf) was estimated using kernel density estimation (for more information about kernel density estimation see [52]).

The marginal posterior distributions were then compared to the prior distributions. The comparison, as also shown in Table 1, reveals a reduction of the variability in most parameters in the posterior distribution. GET and ITT both increase in their mean value but shrink in their CV indicating that the examined population has a delayed and prolonged absorption in comparison to previous studies. The three parameters intP, specCL and specTS, which were uninformed *a priori* in their population mean values (except for physiological constraints), are all informed by the experimental data such that sharp marginal posterior distributions are obtained. Nevertheless, the posterior population variability of intP has a very large coefficient of variation (CV), which indicates large interindividual variability in this parameter. Here, further studies could try to investigate if such large variability may be unphysiological and whether absolute constraints should have been set narrower.

**Table 1:** Comparison of characteristic parameters of the prior and posterior population distributions. Prior and posterior geometric mean values and coefficients of variations (CV) are shown for nine exemplary physiological parameters.

	prior		posterior	
	geometric mean val.	CV [%]	geometric mean val.	CV [%]
**intP [dm/min]**	3.68E-06	2841	1.76E-06	222
**specCL [1/min]**	0.05	204	0.017	26
**specTS [1/min]**	0.02	206	0.071	30
**GET [min]**	17.25	31	24.46	14
**ITT [min]**	146.4	28	183.68	18
**fat vol. [L]**	14.98	65	14.49	46
**kidney vol. [L]**	0.44	30	0.34	25
**liver vol. [L]**	2.36	28	2.1	30
**muscle vol. [L]**	32.099	26	26.35	11

**Fig 5:**
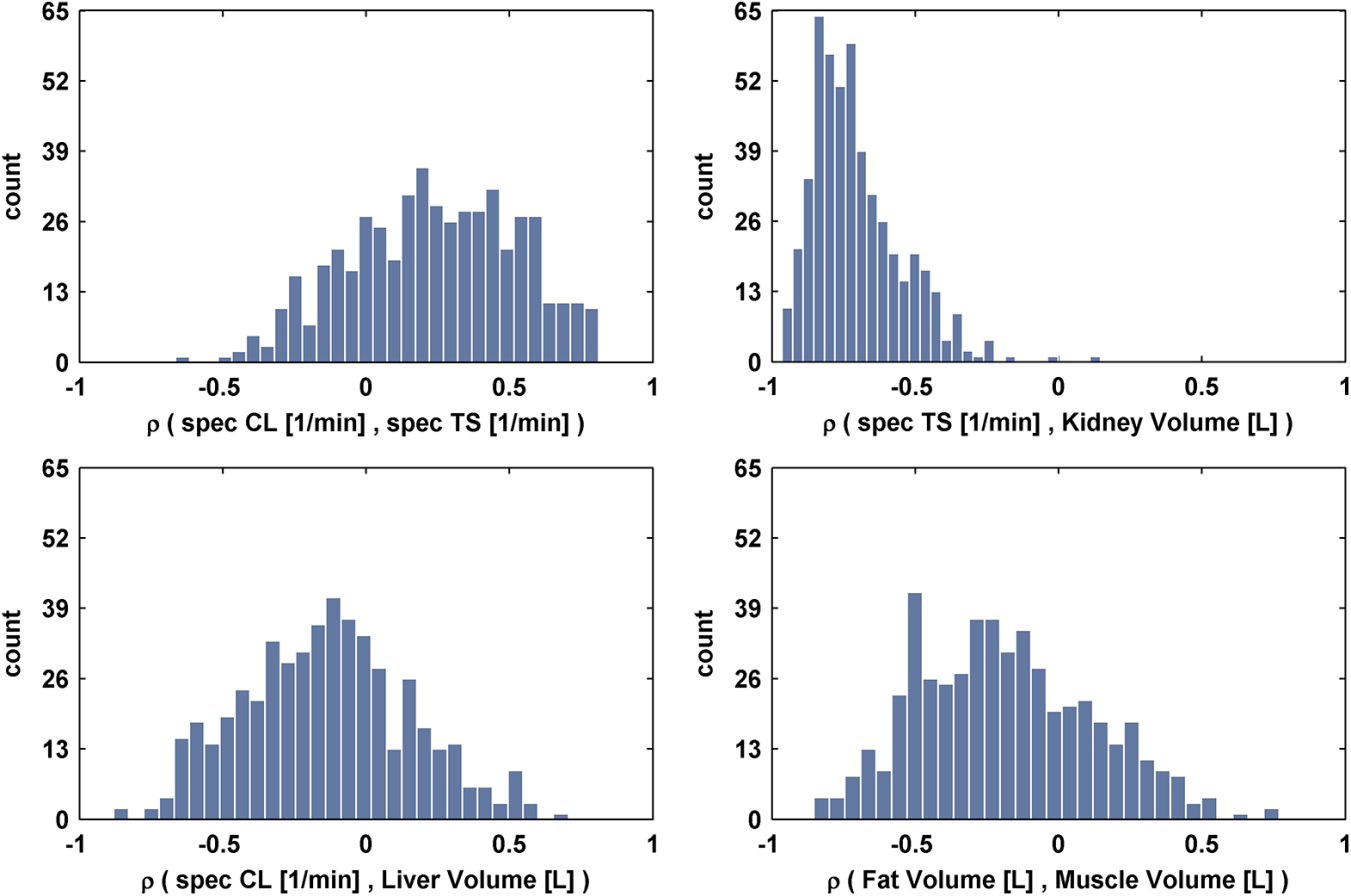
Exemplary representation of derived distributions of correlation between the population parameters. The correlation of a pair of parameters along all individuals was calculated for each of the 500 subsamples of the posterior distribution. For each pair of parameters the histogram of all correlations is shown, representing the uncertainty of the respective correlation.

To investigate possible dependencies in the high dimensional posterior population distribution, a correlation analysis was performed. Thereby, the correlation matrix of all individual parameters was calculated along the individuals to estimate the parameter dependencies within the population as described in Eqs (10) and (11). To account for the uncertainty within this analysis, the correlation matrix was calculated for each of the 500 subsamples out of the posterior distribution. Fig 5 shows the distribution of correlations between exemplary parameters. In particular, a significant mean negative correlation of about 70% (p < 0.05, mean confidence interval [-0.85–0.49]) between specTS and kidney volume can be obtained. The other three exemplary distributions do not have a significant mean correlations; however, a mean positive correlation of about 30% (p > 0.05, mean confidence interval [-0.31 0.70]) can be obtained between specCL and specTS. No further significant correlations could be found after performing a complete analysis of all possible individual parameter combinations (results not shown). Notably, such correlations are derived despite the prior assumption of independent random effects as described in Eq 3. Furthermore, the distinction between individual level and population level is important for the correct meaning of parameter correlations. For population simulations, parameter correlations along individuals are important since such correlations need to be included for the parameterization of a new individual. For model characterization, the aforementioned correlations of individual parameters along the samples of the Markov chain are important. For example, a significant positive correlation of about 42% can be identified for intP and GET (p < 0.05) along the autocorrelation-free samples. Such dependencies denote an essential part of intraindividual uncertainty and need consideration when simulating individual PK profiles as demonstrated in Fig 2.

### Visual predictive check of population pharmacokinetics

Next, a visual predictive check (VPC) was performed with the assessed posterior distribution of the population parameters, to verify the estimated posterior dependency structure of the random effects and our estimated interindividual variability [53]. The VPC diagnoses the fixed effects as well as the random effects by calculating the confidence intervals of the median and the 5% and 95% percentile of a large amount of simulations of the experimental setting. This was important to interpret objectively if the simulations with the posterior results describe the data well.

**Fig 6:**
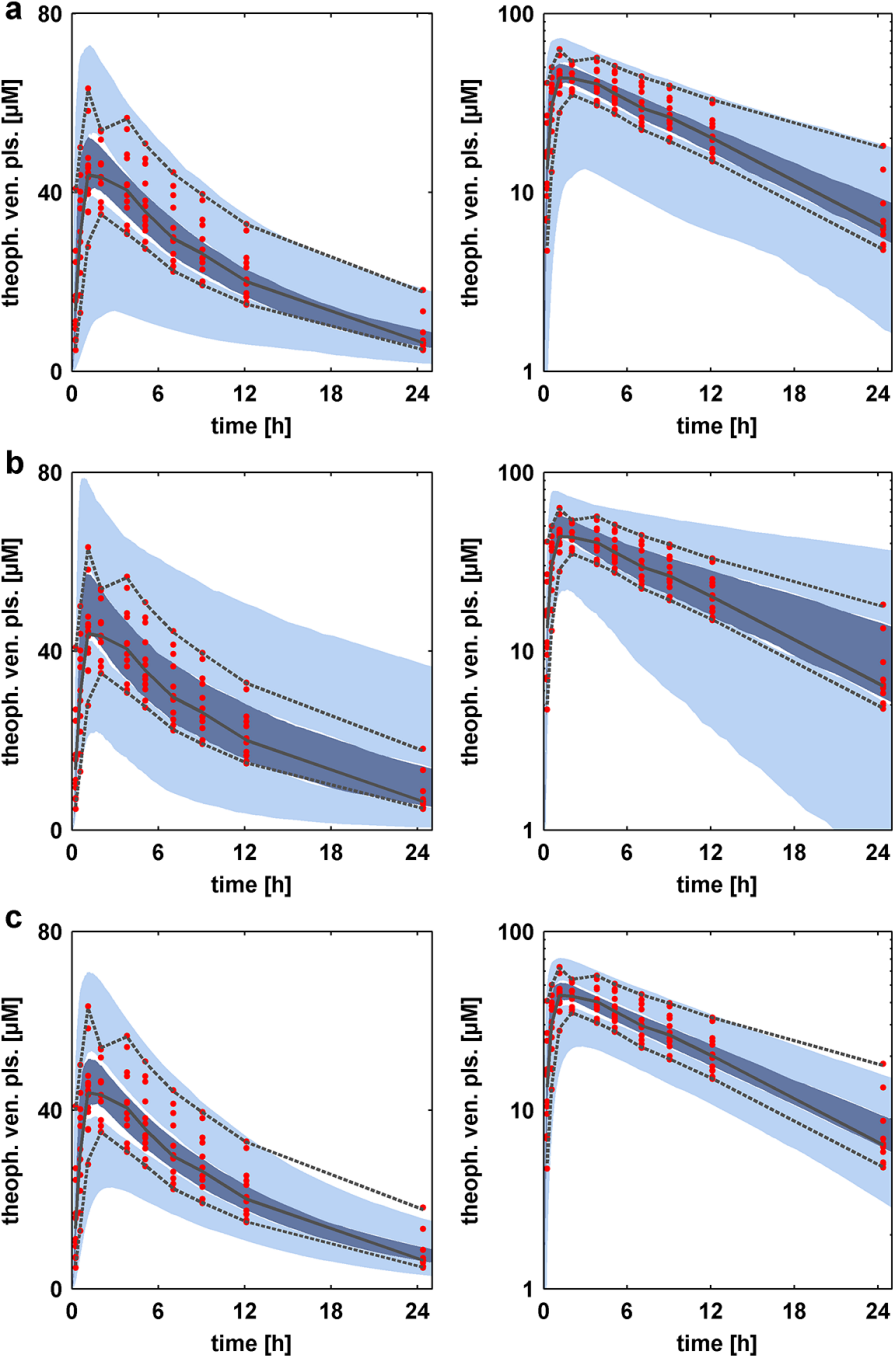
Comparison of visual predictive checks of population pharmacokinetics. (**a**) Visual predicive check (VPC) of the pharmacokinetic behavior using the posterior distributions based on the presented Bayesian population PBPK approach. The uncertainty in population parameters was included in the VPC. (**b**) Visual predicive check of the pharmacokinetic behavior using the prior distributions of all parameters. (**c**) Visual predicive check (VPC) of the pharmacokinetic behavior using the maximum posterior estimates of the posterior distribution based on the presented Bayesian population PBPK approach. Each VPC is presented in linear scale (left) and logarithmic scale (right). The VPCs were performed as described in the text. In each VPC, the 5% and 95% percentiles (black dotted lines) and the median (black line) of the experimental data (red dots) are compared against the 95% confidence intervals of the 5% and 95% percentile of the simulation (light blue area) and the median (blue area).

For each individual, 500 parameters sets were randomly drawn from the full posterior distribution using the multivariate approach in Eqs (10–12). Next, 500 simulations were performed with the generated parameter sets, whereby one simulation consisted of the simulation of all individuals. Fig 6A depicts the resulting 95% confidence intervals for the median and the 5% and 95% percentiles in linear and logarithmic scale, respectively. In addition, the experimental data together with the corresponding median and percentiles are illustrated. The confidence interval of the simulation medians fits the median of the experimental data well, indicating a good identification of the parameter distributions and a good estimation of the covariance structure. In addition, the confidence interval of the simulation medians is relatively narrow. In contrast, the confidence intervals of the percentiles are wider and slightly underestimate the percentiles of the experimental data.

A second VPC was then performed using only prior information to compare against the VPC using the presented Bayesian population PBPK approach. The parameter distributions that need to be considered for the approach consist of the prior knowledge that was integrated into our population PBPK approach. For the three uninformed parameters intP, specCL and specTS a lognormal distribution with a geometric standard deviation of 1.5 was considered. That should provide reasonable values regarding to the literature [18, 54]. The mean value was assumed to be the respective parameter value of the adjusted mean value model. The resulting confidence intervals are shown in Fig 6B and show considerably different results. Especially in the terminal phase (time > 12 h), the confidence interval of the 95% percentile is much wider than the one in Fig 6A. More variability can also be observed when comparing the confidence intervals of the median. With regard to the experimental data, the simulated confidence interval of the 95% percentile overestimates the 95% percentile of the experimental data. The confidence interval of the median overestimates the data in the absorption phase but fits the experimental data well in the elimination phase.

As described above, the generation of new parameter sets out of the posterior takes the inferred uncertainty of the population mean value and the population standard deviation into account. Often, maximum posterior estimates of the population parameters are considered for analyses with the posterior distribution [22, 53, 55]. Fig 6C depicts a VPC based on the posterior estimates of the population parameters. The posterior estimate was chosen as the parameter set with the highest posterior probability. The results show a very good agreement to the experimental data. Especially the confidence interval of the 5% percentile is in better agreement with the data than in Fig 6A and 6B.

### Positive control

In addition, we performed a positive control run to further validate our approach. By sampling from the defined prior distributions (for the population mean values of intP, spec CL and spec TS we used normal distributed priors) for the population mean values and standard deviations we generated a dataset of 12 individuals from the population distribution. We put noise on the data to simulate a relative measurement error of 10%. We run the approach for 150,000 iterations. The first 50,000 iterations were discarded as burn-in, the resulting 100,000 iterations were converged with a maximum Gelman-Rubin criterion of 1.05. Result analyses were performed as described above. No appreciable deviations between prior and posterior could be observed (S5 Table). No significant correlations were obtained. The VPC considering the maximum posterior estimates showed a very good agreement to the experimental data (S3 Fig).

## Discussion

In this work, a combined Bayesian population PBPK approach is presented for the characterization and identification of physiologically-realistic interindividual variability. The approach consists of an hierarchical model, which describes the experimental data at an individual level and at the same time identifies the variability in physiological parameters at the population level. By use of a highly detailed mechanistic PBPK model the interindividual variability in specific parameters can be assigned to a physiological process, such as time of intestinal transit or the volume of a specific organ. The incorporation of the model approach in a Bayesian framework allows integrating large amounts of prior knowledge about the population’s physiology. The Bayesian population PBPK approach calibrates such knowledge to the experimental data and the resulting posterior distribution contains the full information about all parameters and their variation in the population.

The application example simulating theophylline PK revealed the variety of information that is possible to obtain by using the approach. As a first result, individual-specific simulations under consideration of assessed parameter uncertainty described the experimental data in good agreement and with little uncertainty on the PK level. Such information can be considered e.g. to identify possible heterogeneities in the population or even to identify subgroups related to genetic or physiological differences or diseases [56].

Furthermore, a comparison of marginal population distributions of the posterior against the prior distributions demonstrated, which information can be inferred by the experimental data. Since the experimental data represented the PK of a very homogenous population related to their physiology, several population parameter distributions showed a decrease of their variance. Additionally, changes in the mean values indicated a different behavior of the drug, for example in the absorption processes. In particular, theophylline seemed to be absorbed more slowly, which was represented by prolonged GET and ITT. A possible reason could be that the individuals have not been in fasted state during the experimental investigation. Moreover, the posterior population distributions of the organ volumes suggested a lower average body weight of the individuals, since e.g. muscle volume decreases in its mean value and the body weight is composed of the organ volumes in the PBPK model [15].

However, simple observation of the marginal distributions is insufficient to thoroughly analyze the posterior distribution. Only the investigation of the complete high dimensional posterior distribution provides the full information, at least if the prior assumption of independence between parameters cannot be assumed anymore. The illustrated distributions of correlations demonstrated that the assumption of independence between the parameters does not hold and information about the model and the physiology of the population can only be described by the complete multivariate posterior. This can be seen as a natural consequence of incorporation of a physiologically-realistic PBPK model into the Bayesian framework, since effective parameters like a total clearance rate are divided into physiological relationships of e.g. catalytic constants of enzymes and the corresponding volume of an organ. However it has to be noted though that large uncertainty could be observed on the estimated correlations. A possible reason could be the small sample size of 12 individuals that have been considered for the determination of the correlations. Here, investigations that integrate a larger population would deliver more precise results regarding the correlations.

The findings of a dependent multivariate posterior distribution were further supported by consideration of the performance of the VPCs. The use of independent *a priori* information (Fig 6B) of the parameters led to a too large interindividual variability and would provide vague information about the PK behavior of theophylline in a homogenous population. The Bayesian population PBPK approach (Fig 6A) provided a smaller interindividual variability especially in the mean value, and the shape of the intervals was in better agreement with the experimental data. Hence, the VPC also served as a validation for the estimated high dimensional posterior distribution of the population parameters.

The final comparison of a VPC based on the maximum posterior estimate (Fig 6C) and the VPC based on the full posterior information revealed the influence of parameter uncertainty on the population level. Due to the small population that was investigated in our application, large uncertainty is expected in the identified population distribution. Such uncertainty should also be taken into account in model extrapolations; otherwise the extrapolations could underestimate the interindividual variability. Nevertheless, for validation of our approach, the maximum posterior estimate more appropriately demonstrates the successful estimation of the population distributions.

Notably, the assumption of a multivariate normal distribution for the estimation of the dependency structure of the random effects could also be crosschecked against other dependency structures, such as copulas. However, we do not expect large variations in the effect of different dependency structures, since only one significant correlation was identified and all correlations were afflicted with large uncertainties. Nevertheless, the analyses revealed the existence of correlations between population parameters.

The additional consideration of a positive control run demonstrated the validity of the presented results obtained from our Bayesian population PBPK approach. With our approach we successfully extracted information out of the experimental data to update the predefined prior distributions. When no information was included by the experimental data as tested in the positive control, the posterior distributions reflected only the defined prior knowledge.

In classical PopPK, various additional covariates are tested during model identification, such as body weight or creatinine clearance. In this work we used an approach by Willmann et al [15], where age, gender and height were chosen as covariates to create a large physiological database. We used this database for definition of our prior distributions and to implement the covariate approach. This is in line with [32], who demonstrated that the organ weights were better correlated with body height than with BMI or BW. However, other approaches show contrary results and could be tested in future studies to use e.g. lean body mass as covariates [57]. For the theophylline dataset used in our approach, in a PopPK approach Tornøe et al. showed that body weight is no significant covariate [3], however, body weight has also been shown to be a covariate of e.g. clearance in markedly obese patients [58, 59].

All in all, the here presented Bayesian population PBPK approach identifies the interindividual variability in the PK of a population and thereby also characterizes the interindividual variability in the underlying physiological and ADME-related parameters as well as the drug-specific parameters. The resulting high dimensional parameter distribution can be described well via a multivariate normal distribution, which integrates covariance information between physiological parameters that are needed to thoroughly describe the PK behavior of the population. The highly-detailed mechanistic PBPK model offers very good extrapolation capacities, since the derived physiological parameter distribution is easily transferable to other investigations with the same population, where it can be used as new prior information. Further applications of this approach therefore arise in the characterization of special populations where only very sparse data is available. Due to the generalized PBPK model the approach can be executed iteratively and therefore is able to calibrate the posterior distribution over several clinical studies, which will sharpen predictions of the PK behavior of new drugs in special disease states or elderly or pediatric populations.

## Supporting Information

S1 Fig Experimental data of theophylline pharmacokinetics. (PDF)

S2 Fig Individual-specific model simulations of theophylline venous plasma concentrations in linear scale. (PDF)

S3 Fig Visual predicive check (VPC) of the pharmacokinetic behavior using the posterior distributions of the positive control run based on the presented Bayesian population PBPK approach. (PDF)

S1 Table Varied individual parameters together with start value and parameter constraints of the final MCMC run. (PDF)

S2 Table Experimental data of theophylline pharmacokinetics. (PDF)

S3 Table Anthropometric parameters of the considered cohort of individuals. (PDF)

S4 Table Gelman and Rubin convergence criterion. (PDF)

S5 Table Comparison of prior and posterior geometric mean values and coefficients of variations (CV) for nine exemplary physiological parameters of the positive control run. (PDF)
