# Supplementary material for: Bayesian Population Physiologically-Based Pharmacokinetic (PBPK) Approach for a Physiologically Realistic Characterization of Interindividual Variability in Clinically Relevant Populations
Source: PLoS One. 2015 Oct 2;10(10):e0139423. doi: 10.1371/journal.pone.0139423 (PMC4592188; doi:10.1371/journal.pone.0139423)
Supplement: S1 Fig — (PDF) [file pone.0139423.s001.pdf]

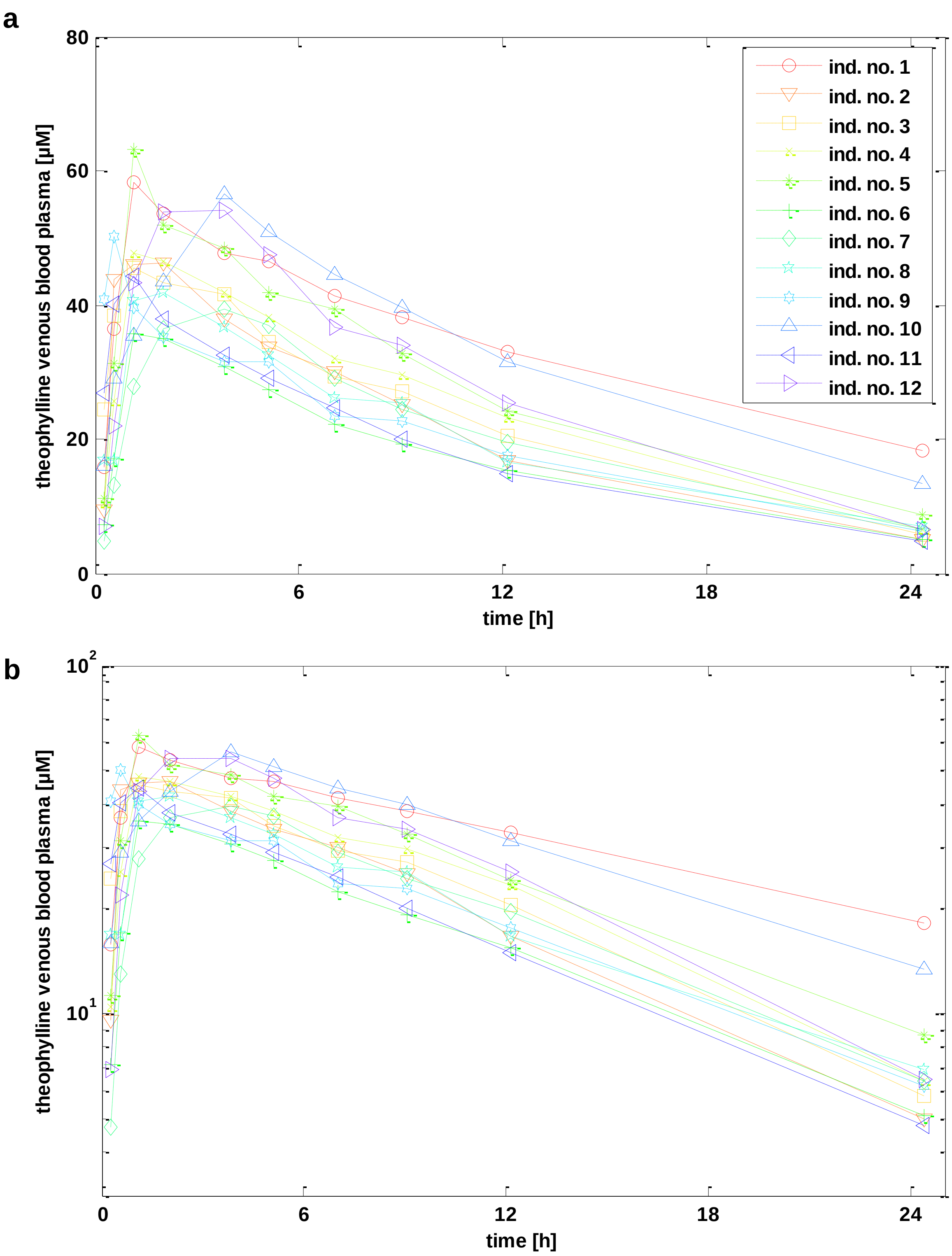

Figure S1: Experimental data of theophylline pharmacokinetics. The individual data is also provided in S2 Table. (**a**) Experimental data on linear scale. (**b**) Experimental data on semi-log scale.
