# Supplementary material for: Bayesian Population Physiologically-Based Pharmacokinetic (PBPK) Approach for a Physiologically Realistic Characterization of Interindividual Variability in Clinically Relevant Populations
Source: PLoS One. 2015 Oct 2;10(10):e0139423. doi: 10.1371/journal.pone.0139423 (PMC4592188; doi:10.1371/journal.pone.0139423)
Supplement: S2 Fig — (PDF) [file pone.0139423.s002.pdf]

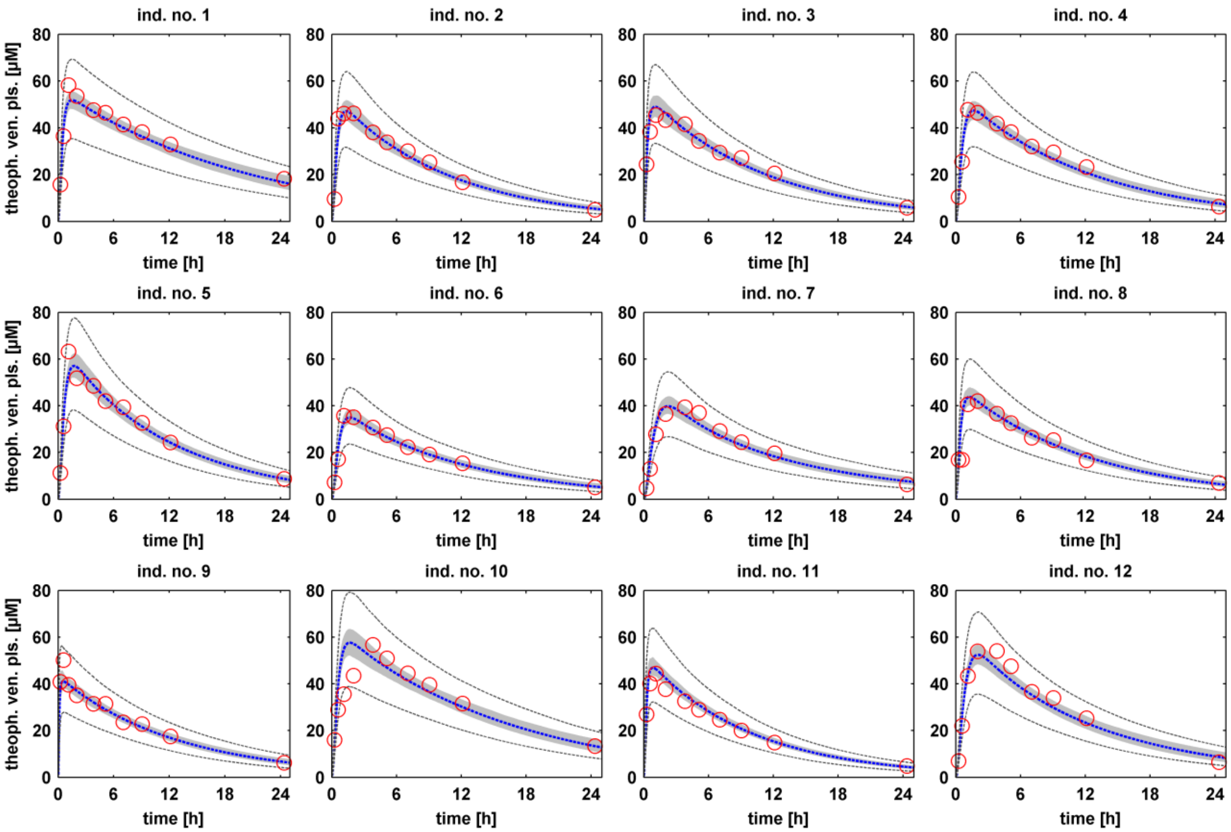

Figure S2: Individual-specific model simulations of theophylline venous plasma concentrations in linear scale. For each of the 12 individuals the PBPK model was subsequently parameterized and simulated with each of 500 individual and independent parameter vectors out of the posterior distribution. The 95 % confidence interval of all simulations (grey area) is shown together with the mean value curve (blue dotted line) and the experimental data (red circles). Dark grey dotted lines depict the upper and lower bound of the 95 % confidence interval of all simulations including the inferred measurement error.
