# Supplementary material for: Bayesian Population Physiologically-Based Pharmacokinetic (PBPK) Approach for a Physiologically Realistic Characterization of Interindividual Variability in Clinically Relevant Populations
Source: PLoS One. 2015 Oct 2;10(10):e0139423. doi: 10.1371/journal.pone.0139423 (PMC4592188; doi:10.1371/journal.pone.0139423)
Supplement: S3 Fig — (PDF) [file pone.0139423.s003.pdf]

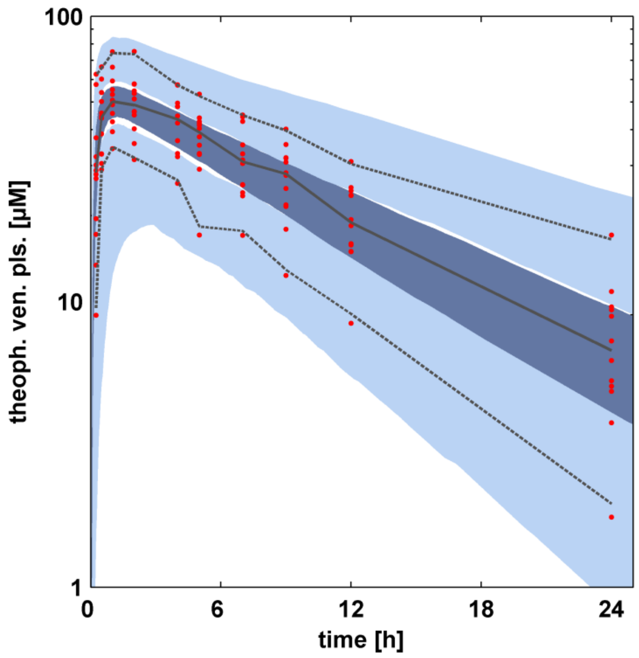

Figure S3: Visual predicive check (VPC) of the pharmacokinetic behavior using the posterior distributions of the positive control run based on the presented Bayesian population PBPK approach. The maximum posterior estimates of the population parameters have been used to estimate the posterior population distributions. The VPCs were performed as described in the text. In each VPC, the 5 % and 95 % percentiles (black dotted lines) and the median (black line) of the experimental data (red dots) are compared against the 95 % confidence intervals of the 5 % and 95 % percentile of the simulation (light blue area) and the median (blue area).
