# Supplementary material for: Bayesian Population Physiologically-Based Pharmacokinetic (PBPK) Approach for a Physiologically Realistic Characterization of Interindividual Variability in Clinically Relevant Populations
Source: PLoS One. 2015 Oct 2;10(10):e0139423. doi: 10.1371/journal.pone.0139423 (PMC4592188; doi:10.1371/journal.pone.0139423)
Supplement: S1 Table — (PDF) [file pone.0139423.s004.pdf]

Table S1: Varied individual parameters together with start value and parameters constraints (min., max.) of the final MCMC run. Start values are exemplary for individual 1, start values for the other individuals differ slightly.

Name	Type	Unit	Start value	Min. value	Max. value	Prior distribution
Intestinal permeability	Individual	dm/min	1.32E-06	3.57E-08	3.57E-04	Lognormal
Hepatic clearance constant	Individual	1/min	9.31E-03	5.00E-03	5.00E-01	Lognormal
Renal clearance constant	Individual	1/min	3.17E-02	2.00E-03	2.00E-01	Lognormal
Stomach gastric emptying time	Individual	min	29.333	10.000	30.000	Lognormal
Small intestinal transit time	Individual	min	199.070	90.000	240.000	Lognormal
Plasma protein scale factor	Individual	-	0.844	0.700	1.300	Lognormal
Venous Blood volume	Individual	L	1.020	0.763	1.174	Normal
Arterial Blood volume	Individual	L	0.462	0.332	0.510	Normal
Bone specific blood flow rate	Individual	L/min/kg organ	0.026	0.023	0.032	Normal
Bone volume	Individual	L	12.675	9.443	14.230	Normal
Brain volume	Individual	L	1.687	1.282	1.735	Normal
Brain specific blood flow rate	Individual	L/min/kg organ	0.566	0.439	0.594	Normal
Fat volume	Individual	L	22.422	3.641	58.480	Lognormal
Fat specific blood flow rate	Individual	L/min/kg organ	0.020	0.019	0.025	Normal
Gonads volume	Individual	L	0.040	0.032	0.049	Normal
Gonads specific blood flow rate	Individual	L/min/kg organ	0.074	0.069	0.093	Normal
Heart volume	Individual	L	0.341	0.165	0.696	Normal
Heart specific blood flow rate	Individual	L/min/kg organ	0.594	0.530	0.717	Normal
Kidney volume	Individual	L	0.425	0.106	0.806	Normal

Kidney specific blood flow rate	Individual	L/min/kg organ	2.897	2.573	3.481	Normal
Stomach volume	Individual	L	0.153	0.086	0.259	Normal
Stomach specific blood flow rate	Individual	L/min/kg organ	0.366	0.328	0.444	Normal
Small intestine volume	Individual	L	0.622	0.438	1.036	Normal
Small intestine specific blood flow rate	Individual	L/min/kg organ	0.849	0.763	1.032	Normal
Large intestine volume	Individual	L	0.445	0.159	0.693	Normal
Large intestine specific blood flow rate	Individual	L/min/kg organ	0.592	0.536	0.725	Normal
Liver volume	Individual	L	0.837	0.655	4.249	Normal
Liver specific blood flow rate	Individual	L/min/kg organ	0.171	0.152	0.206	Normal
Lung volume	Individual	L	1.166	0.549	3.012	Lognormal
Muscle volume	Individual	L	31.096	20.080	50.163	Lognormal
Muscle specific blood flow rate	Individual	L/min/kg organ	0.038	0.029	0.039	Normal
Pancreas volume	Individual	L	0.184	0.034	0.364	Normal
Pancreas specific blood flow rate	Individual	L/min/kg organ	0.351	0.291	0.393	Normal
Portal vein volume	Individual	L	1.079	0.821	1.263	Normal
Skin volume	Individual	L	4.578	2.745	4.889	Normal
Skin specific blood flow rate	Individual	L/min/kg organ	0.079	0.073	0.099	Normal
Spleen volume	Individual	L	0.370	0.067	0.870	Lognormal
Spleen specific blood flow rate	Individual	L/min/kg organ	0.740	0.681	0.921	Normal
Lipophilicity	Global	-	1.380	-1.000	1.500	Uniform
Unbound protein fraction	Global	-	0.355	0.330	0.500	Uniform
Measurement error	Global	-	0.137	0.010	1.000	Jeffreys
