# Supplementary material for: Bayesian Population Physiologically-Based Pharmacokinetic (PBPK) Approach for a Physiologically Realistic Characterization of Interindividual Variability in Clinically Relevant Populations
Source: PLoS One. 2015 Oct 2;10(10):e0139423. doi: 10.1371/journal.pone.0139423 (PMC4592188; doi:10.1371/journal.pone.0139423)
Supplement: S2 Table — (PDF) [file pone.0139423.s005.pdf]

Table S2: Experimental data of theophylline pharmacokinetics. Data are from Boeckmann et al. (Boeckmann et al, 1994) and can be found as 'theoph' data frame in the software package R.

ID	Subject	Body Weight [kg]	Dose [mg/kg]	Time [h]	Concentration [mg/L]
1	1	79.6	4.02	0	0.74
2	1	79.6	4.02	0.25	2.84
3	1	79.6	4.02	0.57	6.57
4	1	79.6	4.02	1.12	10.5
5	1	79.6	4.02	2.02	9.66
6	1	79.6	4.02	3.82	8.58
7	1	79.6	4.02	5.1	8.36
8	1	79.6	4.02	7.03	7.47
9	1	79.6	4.02	9.05	6.89
10	1	79.6	4.02	12.12	5.94
11	1	79.6	4.02	24.37	3.28
12	2	72.4	4.4	0	0
13	2	72.4	4.4	0.27	1.72
14	2	72.4	4.4	0.52	7.91
15	2	72.4	4.4	1	8.31
16	2	72.4	4.4	1.92	8.33
17	2	72.4	4.4	3.5	6.85
18	2	72.4	4.4	5.02	6.08
19	2	72.4	4.4	7.03	5.4
20	2	72.4	4.4	9	4.55
21	2	72.4	4.4	12	3.01
22	2	72.4	4.4	24.3	0.9
23	3	70.5	4.53	0	0
24	3	70.5	4.53	0.27	4.4
25	3	70.5	4.53	0.58	6.9
26	3	70.5	4.53	1.02	8.2
27	3	70.5	4.53	2.02	7.8
28	3	70.5	4.53	3.62	7.5
29	3	70.5	4.53	5.08	6.2
30	3	70.5	4.53	7.07	5.3
31	3	70.5	4.53	9	4.9
32	3	70.5	4.53	12.15	3.7
33	3	70.5	4.53	24.17	1.05
34	4	72.7	4.4	0	0
35	4	72.7	4.4	0.35	1.89
36	4	72.7	4.4	0.6	4.6
37	4	72.7	4.4	1.07	8.6
38	4	72.7	4.4	2.13	8.38
39	4	72.7	4.4	3.5	7.54

40	4	72.7	4.4	5.02	6.88
41	4	72.7	4.4	7.02	5.78
42	4	72.7	4.4	9.02	5.33
43	4	72.7	4.4	11.98	4.19
44	4	72.7	4.4	24.65	1.15
45	5	54.6	5.86	0	0
46	5	54.6	5.86	0.3	2.02
47	5	54.6	5.86	0.52	5.63
48	5	54.6	5.86	1	11.4
49	5	54.6	5.86	2.02	9.33
50	5	54.6	5.86	3.5	8.74
51	5	54.6	5.86	5.02	7.56
52	5	54.6	5.86	7.02	7.09
53	5	54.6	5.86	9.1	5.9
54	5	54.6	5.86	12	4.37
55	5	54.6	5.86	24.35	1.57
56	6	80	4	0	0
57	6	80	4	0.27	1.29
58	6	80	4	0.58	3.08
59	6	80	4	1.15	6.44
60	6	80	4	2.03	6.32
61	6	80	4	3.57	5.53
62	6	80	4	5	4.94
63	6	80	4	7	4.02
64	6	80	4	9.22	3.46
65	6	80	4	12.1	2.78
66	6	80	4	23.85	0.92
67	7	64.6	4.95	0	0.15
68	7	64.6	4.95	0.25	0.85
69	7	64.6	4.95	0.5	2.35
70	7	64.6	4.95	1.02	5.02
71	7	64.6	4.95	2.02	6.58
72	7	64.6	4.95	3.48	7.09
73	7	64.6	4.95	5	6.66
74	7	64.6	4.95	6.98	5.25
75	7	64.6	4.95	9	4.39
76	7	64.6	4.95	12.05	3.53
77	7	64.6	4.95	24.22	1.15
78	8	70.5	4.53	0	0
79	8	70.5	4.53	0.25	3.05
80	8	70.5	4.53	0.52	3.05
81	8	70.5	4.53	0.98	7.31
82	8	70.5	4.53	2.02	7.56

83	8	70.5	4.53	3.53	6.59
84	8	70.5	4.53	5.05	5.88
85	8	70.5	4.53	7.15	4.73
86	8	70.5	4.53	9.07	4.57
87	8	70.5	4.53	12.1	3
88	8	70.5	4.53	24.12	1.25
89	9	86.4	3.1	0	0
90	9	86.4	3.1	0.3	7.37
91	9	86.4	3.1	0.63	9.03
92	9	86.4	3.1	1.05	7.14
93	9	86.4	3.1	2.02	6.33
94	9	86.4	3.1	3.53	5.66
95	9	86.4	3.1	5.02	5.67
96	9	86.4	3.1	7.17	4.24
97	9	86.4	3.1	8.8	4.11
98	9	86.4	3.1	11.6	3.16
99	9	86.4	3.1	24.43	1.12
100	10	58.2	5.5	0	0.24
101	10	58.2	5.5	0.37	2.89
102	10	58.2	5.5	0.77	5.22
103	10	58.2	5.5	1.02	6.41
104	10	58.2	5.5	2.05	7.83
105	10	58.2	5.5	3.55	10.21
106	10	58.2	5.5	5.05	9.18
107	10	58.2	5.5	7.08	8.02
108	10	58.2	5.5	9.38	7.14
109	10	58.2	5.5	12.1	5.68
110	10	58.2	5.5	23.7	2.42
111	11	65	4.92	0	0
112	11	65	4.92	0.25	4.86
113	11	65	4.92	0.5	7.24
114	11	65	4.92	0.98	8
115	11	65	4.92	1.98	6.81
116	11	65	4.92	3.6	5.87
117	11	65	4.92	5.02	5.22
118	11	65	4.92	7.03	4.45
119	11	65	4.92	9.03	3.62
120	11	65	4.92	12.12	2.69
121	11	65	4.92	24.08	0.86
122	12	60.5	5.3	0	0
123	12	60.5	5.3	0.25	1.25
124	12	60.5	5.3	0.5	3.96
125	12	60.5	5.3	1	7.82

126	12	60.5	5.3	2	9.72
127	12	60.5	5.3	3.52	9.75
128	12	60.5	5.3	5.07	8.57
129	12	60.5	5.3	7.07	6.59
130	12	60.5	5.3	9.03	6.11
131	12	60.5	5.3	12.05	4.57
132	12	60.5	5.3	24.15	1.17

References

Boeckmann A, Sheiner L, Beal S (1994) NONMEM Users Guide: Part V. University of California, San Francisco,
