# Supplementary material for: Bayesian Population Physiologically-Based Pharmacokinetic (PBPK) Approach for a Physiologically Realistic Characterization of Interindividual Variability in Clinically Relevant Populations
Source: PLoS One. 2015 Oct 2;10(10):e0139423. doi: 10.1371/journal.pone.0139423 (PMC4592188; doi:10.1371/journal.pone.0139423)
Supplement: S3 Table — (PDF) [file pone.0139423.s006.pdf]

Table S3: Anthropometric parameters of the considered cohort of individuals. Age was considered to be 30 years for all individuals. Body Height was considered to be different from individual to individual. Dose and body weight are described in the used dataset (1) and in Table S2.

ID	Population	Gender	Age [years]	Body Weight [kg]	Height [cm]	BMI	Dose [mg/kg]	total dose [mg]
individual 1	European	male	30.00	79.60	182	24.03	4.02	319.99
individual 2	European	male	30.00	72.40	173	24.19	4.40	318.56
individual 3	European	male	30.00	70.50	189	19.74	4.53	319.37
individual 4	European	male	30.00	72.70	180	22.44	4.40	319.88
individual 5	European	male	30.00	54.60	160	21.33	5.86	319.96
individual 6	European	male	30.00	80.00	183	23.89	4.00	320.00
individual 7	European	male	30.00	64.60	184	19.08	4.95	319.77
individual 8	European	male	30.00	70.50	185	20.60	4.53	319.37
individual 9	European	male	30.00	86.40	190	23.93	3.10	267.84
individual 10	European	male	30.00	58.20	167	20.87	5.50	320.10
individual 11	European	male	30.00	65.00	176	20.98	4.92	319.80
individual 12	European	male	30.00	60.50	174	19.98	5.30	320.65

References

(1) Boeckmann, A., Sheiner, L. & Beal, S. *NONMEM Users Guide: Part V* (University of California, San Francisco: 1994).
