# Supplementary material for: Bayesian Population Physiologically-Based Pharmacokinetic (PBPK) Approach for a Physiologically Realistic Characterization of Interindividual Variability in Clinically Relevant Populations
Source: PLoS One. 2015 Oct 2;10(10):e0139423. doi: 10.1371/journal.pone.0139423 (PMC4592188; doi:10.1371/journal.pone.0139423)
Supplement: S4 Table — (PDF) [file pone.0139423.s007.pdf]

Table S4: Gelman and Rubin convergence criterion. Gelman and Rubin convergence criterion was determined after splitting the Markov chain into two chains with the same length. $\hat{R}$ for individual parameters represents the mean value of all 12 individuals. For parameters with a high $\hat{R}$, convergence was calculated in addition for the Markov chain representing the functional relationship (see hepatic clearance x liver volume and kidney clearance x kidney volume). Overall mean value for $\hat{R}$ is 1.14, when taking all 535 parameters into account.

**parameter**	**parameter type**	$\hat{\mathbf{R}}$
Intestinal permeability	individual	1.00
Hepatic clearance constant	individual	1.17
Renal clearance constant	individual	1.74
Stomach gastric emptying time	individual	1.00
Small intestinal transit time	individual	1.00
Plasma protein scale factor	individual	1.01
Fat volume	individual	1.03
Kidney volume	individual	1.98
Liver volume	individual	1.22
Muscle volume	individual	1.02
Hepatic clearance constant x liver volume	individual	1.11
Renal clearance constant x kidney volume	individual	1.01
Lipophilicity	global	1.02
Unbound protein fraction	global	1.03
Intestinal permeability	population	1.63
Hepatic clearance constant	population	3.84
Renal clearance constant	population	2.18
Stomach gastric emptying time	population	1.00
Small intestinal transit time	population	1.01
Plasma protein scale factor	population	1.01
Fat volume	population	1.02
Kidney volume	population	2.33
Liver volume	population	1.54
Muscle volume	population	1.04
Hepatic clearance constant x liver volume	population	1.03
Renal clearance constant x kidney volume	population	1.05
