# Supplementary material for: Bayesian Population Physiologically-Based Pharmacokinetic (PBPK) Approach for a Physiologically Realistic Characterization of Interindividual Variability in Clinically Relevant Populations
Source: PLoS One. 2015 Oct 2;10(10):e0139423. doi: 10.1371/journal.pone.0139423 (PMC4592188; doi:10.1371/journal.pone.0139423)
Supplement: S5 Table — (PDF) [file pone.0139423.s008.pdf]

**Table S5: Comparison of prior and posterior geometric mean values and coefficients of variations (CV) for nine exemplary physiological parameters of the positive control run.**

| | prior | | posterior | |
|---|---|---|---|---|
| | geometric mean val. | CV [%] | geometric mean val. | CV [%] |
| intP [dm/min] | 1.73E-06 | 217 | 1.77E-06 | 219 |
| specCL [1/min] | 0.017 | 23 | 0.018 | 21 |
| specTS [1/min] | 0.071 | 19 | 0.07 | 16 |
| GET [min] | 25.65 | 15 | 27.72 | 13 |
| ITT [min] | 194.2 | 19 | 208.14 | 16 |
| fat vol. [L] | 14.83 | 42 | 15.43 | 36 |
| kidney vol. [L] | 0.44 | 25 | 0.41 | 21 |
| liver vol. [L] | 2.36 | 23 | 2.33 | 21 |
| muscle vol. [L] | 32.29 | 10 | 31.97 | 9 |
